# Old principles, persisting challenges: Maternal health care market alignment in Mexico in the search for UHC

**DOI:** 10.1371/journal.pone.0199543

**Published:** 2018-07-02

**Authors:** Roxana Rodríguez-Franco, Edson Serván-Mori, Octavio Gómez-Dantés, David Contreras-Loya, Carlos Pineda-Antúnez

**Affiliations:** 1 National Institute of Public Health, Cuernavaca, Morelos, Mexico; 2 University of California, Berkeley, California, United States of America; Seoul National University College of Medicine, REPUBLIC OF KOREA

## Abstract

The purpose of this study is to analyze the alignment of supply and demand for antenatal care (ANC) in Mexico based on the definition of access provided by Donabedian: the “degree of adjustment” between resources and needs. Alignment was studied in the teenage and adult population of Mexico that lacked conventional social security between 2008 and 2015, a period of expanding financial resources for health and public health insurance coverage. Spatial econometric methods were used to analyze data from the Ministry of Health on the supply and demand for ANC in 2,314 municipalities (94% of all municipalities in Mexico). During this period, the relative weight of ANC demand among adolescents increased 37% while the production of antenatal consultations for adolescent and adult women remained unchanged. Bivariate spatial analyses of correlation between supply and demand for ANC services yielded a minimal spatial correlation, or lack of territorial correspondence, between supply and demand among women in both age groups. Spatial econometric analysis confirmed a non-significant association between supply and demand for ANC services. Our findings suggest the existence of misalignment between supply and demand for these services. This requires a reassessment of the management and delivery of ANC services at the local level in order to increase effective coverage and improve the overall performance of the health system.

## Introduction

The satisfaction of health needs of a population is highly dependent on the way in which health service suppliers and demanders interact [1]. Needs are met when supply and demand are aligned, that is, when health care resources are adequate, sufficient, and available when they are required [2,3].

Donabedian defines health service accessibility as “the degree of adjustment between the characteristics of a population and those of its health services” [4]. According to this definition, health care services are accesible if their characteristicsꟷgeographic availability, organization, price, acceptabilityꟷallow persons to reach them, enter theg facilities in which they are provided, and use them [5]. It follows then that misalignment between supply and demand for services derive in sub-optimal utilization and, eventually, in poor health outcomes and loss of social wellbeing [6].

So alignment between supply and demand for health services is shaped by the characteristics of both, populations and service providers; for example, the aging of population intensifies the demand for rehabilitation services [7]. Geographic location of health care units, for example, affects both the supply and the demand for services [8–12]. With regard to maternal and child health specifically, it has been observed that geographic barriers to access translate into fewer antenatal control visits and reduce the chances of obtaining timely medical services during childbirth [12–21].

In the past 15 years, Mexico has implemented several initiatives in order to guarantee universal health care coverage (UHC) with financial protection. The key instrument in the search for UHC is a new public insurance scheme called *Seguro Popular de Salud* (SP) or Popular Health Insurance aimed at covering the population that has no access to the services offered by conventional social security institutions. SP is a public and voluntary insurance for the non-salaried population, which includes those working in the informal sector of the economy, the self-employed, and the un-employed. With guaranteed access to a package of around 250 essential services and a package of 60 high-cost interventions, SP enrollment has been expanding to reach 56 million in 2015, supported by a major increase in public expenditure in health and the expansion of the supply of health care inputs and services [22–28]. Since its creation, SP sought to improve the conditions of access and use of maternal health services, guaranteeing ―through its synergy with specific programs such as “Arranque Parejo en la Vida” [29]*― *free access to a package of health services, and strengthening human and material capacities in health care units [23,24,30].

Thanks to this and other initiatives, in the past two decades, Mexico has made significant strides in maternal health: the proportion of births assisted by skilled health personnel has grown and maternal mortality has dropped [31]. However, evidence also indicates that the impact on maternal health has been heterogeneous and territorially concentrated [32–35]. Furthermore, two factors have recently shaken the maternal field: a 52.8% boom in the female population aged 15–49 years during the past twenty years and an upsurge in teenage pregnancies and maternity [36,37]. These elements are increasing the demand for maternal care.

In this study we tried to answer the following question: Are maternal health resources spatially aligned with the healthcare needs of women of reproductive age without social security? If so, the supply of maternal health services should be significantly and positively associated with their demand, whereas a null or negative association would indicate misalignment in the public maternal health care market. Alignment was studied in the teenage and adult population of Mexico that lacked conventional social security between 2008 and 2015, a period of characterized by a major expansion of financial resources for health and public health insurance coverage. We defined suppliers as the government, specifically the MoH, which is the provider of first-time ANC consultations, and demanders as 10-to-54-year-old females without social security who have given birth to at least one live-born child. The municipality was chosen as the geographical space that would serve to identify the socio-demographic characteristics of the population and its interactions with the health care system.

## Material and methods

### Data and variables

Our study encompassed all municipalities in Mexico (n = 2,457) and all females aged 10–54 years who lacked social security (either enrolled in SP or with no health insurance). We analyzed public administrative information from the National Health Information System (SINAIS by its Spanish initials) for the period 2008–2015 [38]. Data were analyzed according to the coding system of the General Directorate for Health Information (DGIS by its Spanish acronym). DGIS codes are available for consultation online (see details at http://www.dgis.salud.gob.mx/contenidos/sinais/s_index.html). SINAIS was created to inform the decisions of policy-makers and researcher. It comprises four sub-systems: (1) population and coverage; (2) health resources at health care facilities; (3) health utilization and services delivered (e.g., antenatal consultations); (4) damage to health and births [39]. Data for our study were drawn primarily from subsystems two and three.

Our dependent variable—supply of ANC services—was measured by the number of first-time ANC consultations delivered during 2015 by each Ministry of Health (Secretaría de Salud or SSa) or States Health Services (Servicios Estatales de Salud or SESA) outpatient facility. Our independent variable—demand for ANC services—was measured by the number of females without conventional social security who had given birth to at least one live-born child. The latter were divided into two age groups: 10–19 years old and 20–54 years old. Assuming that the alignment between supply and demand is not automatic, and in order to capture the demographic evolution of service demand, our independent variable was expressed as the percent change in the period 2000–2015.

Control variables included sociodemographic and development indicators at the municipality level and were associated to supply and demand for health care services [40–43], which were measured for 2000 with the aim of controlling the relationship of interest by relevant characteristics prior to the inception of SP. Data for this variables were drawn from censuses and household income and expenditure surveys published by the National Institute of Statistics, Geography and Informatics of Mexico (INEGI by its Spanish acronym) [44]. To measure the availability of health care resources, we constructed two availability and continuous indexes for the period 2001–2015 using a principal components analysis: (i) hospital/equipment resources and (ii) outpatient ANC services [45]. The first referred to the volume of hospitals, hospital beds, operating rooms, and ultrasound equipment, as well as to the number of physicians and nurses in contact with patients. The second included the number of outpatient consultation units and delivery rooms as well as the number of physicians, nurses, and medical students in contact with patients. In order to capture the expansion of resources availability, both indexes were expressed as the 2001–2015 percent change. Table 1 contains the details of the analyzed variables.

**Table 1:** Analytical variables and sources of information.

Variable	Definition	Source	Year
**Independent**			
Supply of antenatal health services	Number of first-time antenatal consultations produced by outpatient primary-care facilities (expressed as outpatient consultation units)	National Health Information System: sub-system on service production by health service facilities	2015
**Dependent**			
Demand for antenatal health services	Females aged 10–54 years who lacked social security and had given birth to at least one live-born child	Information sub-system on births	2015
**Control**			
Population without social security	Percentage of the Mexican population without social security	Population and Housing Census	2000
Rurality	Average percentage of the Mexican population residing in rural areas	Population and Housing Census	2000
Marginalization index	Numeric value for the global intensity of social marginalization at municipal level	National Population Council	2000
Gini coefficient	A relative measure of the degree of concentration in the distribution of household incomes; serves to detect variances among household strata sorted into deciles	National Institute of Statistics, Geography and Informatics (*INEGI*)—data from the National Household Income and Expenditure Survey (*ENIGH*)	2000
Fertility	Average number of live children born to females aged 12 years and above	National Population Council	2000
Population dispersion	Number of inhabitants per km^2^ per municipality	Population and Housing Census, and data regarding continental surface	2000
Indigenous population	Percentage of the population aged five years and above who spoke an indigenous language	Population and Housing Census	2000
Population without education	Percentage of the population (male and female) aged five years and above who lacked any schooling or had only attained preschool education	Population and Housing Census	2000
Maternal health resources: hospital resources and equipment	Δ% 2001–2015 index of available hospital resources and equipment	Information sub-system on health equipment, human resources and infrastructure	2001–2015
Maternal health resources: outpatient health services	Δ% 2001–2015 index of available outpatient health services	Information sub-system on health equipment, human resources and infrastructure	2001–2015

**Note**: First-time consultation: services provided by health personnel to an individual seeking health care at a health-care unit for the first time as a result of illness or another cause.

The final analytic sample was composed of 2,314 municipalities. We excluded those created after 2000 (4) or those that lacked full data on the indicators of interest (139), with the total loss rate amounting to 5.6%.

### Exploratory analysis

First, we performed an exploratory data analysis (EDA) of our study variables using central tendency and dispersion statistics. Additionally, we mapped quintiles of ANC supply and demand at the municipal level. This was done using Stata MP v13.1 statistical software [46].

We then performed an exploratory spatial data analysis (ESDA) of the dependent and independent variables [47]. Based on the assumption that no previous conceptual structure existed for ANC, our work centered on detecting the presence of spatial autocorrelation or dependence in the data [48]. For this analysis we used a spatial weights matrix which considered the five nearest neighbors (K = 5). We adopted the criterion of lack of contiguity in analyzed observations because it enabled us to capture maximum spatial correlation in the data. The p-value used to assess Moran’s I statistical significance (or presence of spatial autocorrelation) was determined after performing 10,000 shocks with GeoDa statistical software [49].

### Spatial econometric analysis

Traditional analysis of supply and demand alignment considers geographic units as insular, without explicitly assuming that data analyzed are either dependent on their location or independent from what takes place among neighboring localities. Conversely, spatial autocorrelation opens up multidirectional relationships among the units observed: what occurs in one municipality impacts the neighboring municipalities and vice-versa.

The assumption that data analyzed are spatially independent, as proposed by standard econometric methods, leads to biased, inconsistent and inefficient parameter estimates [50]. Spatial econometric methods represent a valuable contribution in this context [51,52]. To demonstrate this, we began by specifying a simple linear regression model based on ordinary least squares (OLSs) for m municipalities:

$$Y_{m}=\alpha i_{m}+X_{m}\beta +\varepsilon_{m},$$ (1)

where Y was the supply of ANC services and X a vector of explanatory variables including the demand indicator; ε was the error term, and β a vector of parameters to be estimated. This specification assumed that the results of various municipalities were independent from one another (E[ε|X] = 0) and that ε denoted a normal distribution with homoscedastic variance and a zero average (ε~N[0,σ^2^I]).

According to this specification, if data analyzed were characterized by spatial autocorrelation, that is, if the municipalities spatially nearest to one another indicated a stronger association than the more distant ones, then it would follow that events in one point in space were functionally linked to those in another. This could occur through Y, X or ε. Solving these problems from a spatial econometric perspective requires the use of a spatial weights matrix or spatial lag matrix (W), defined as a squared non-stochastic matrix where W_ij_ elements reflect the intensity of the interdependence between each pair of geographic units i and j, with W weighting higher values to the nearest municipalities. W can be incorporated as follows:

$$Y_{m}={\rho WY_{m}+{\mathrm{\alpha i}}_{m}+X}_{m}\beta +\theta {\mathrm{WX}}_{m}+\mu_{m},\;\mathrm{with}\;\mu_{m}=\lambda W\mu_{m}+\varepsilon_{m}$$ (2)

If ρ = θ = λ = 0, then specification (1) above would be appropriate. However, if spatial dependence were observed in the data, further specifications would be required, namely a spatial lag model (SLM) if ρ ≠ 0 and θ = λ = 0; a spatial error model (SEM) if λ ≠ 0 and ρ = θ = 0; a spatial Durbin model (SDM) if ρ ≠ 0, θ ≠ 0 and λ = 0; and a spatial auto-regressive moving average model (SARMA) if ρ ≠ 0, λ ≠ 0 and θ = 0. The SDM option offers analytic advantages, as it allows for incorporating global spatial effects by estimating the ρ parameter; local spatial effects, or spatial-peer-effects, under θ; and externalities, or spatial-spillover-effects, in neighboring municipalities. Additionally, the SDM nests the SLM, SEM and WX local effects.

Having completed the ESDA and having corroborated the presence of spatial autocorrelation, we proceeded to select a model for our estimations. The decision required hypothesis testing in ρ, θ and λ. To this end, we adopted a bottom-up approach, from the particular to the general, as proposed by Anselin [49]. In such cases, if testing confirms the existence of a lag or spatial error, then the existence of a SEM nested in the SDM must be corroborated. In the event of a positive result, it is appropriate to use the SDM.

We selected our definitive model using the spatdiag command in the Stata MP v13.1 statistical package. The actual estimation and nesting test were performed through maximum likelihood assessment using the spmlreg and lrtest commands (with k = 5). The SDM proved the most appropriate. S1 and S2 Tables in supporting information show the tests performed to verify goodness of fit, compliance with assumptions, and spatial correlation. The total, direct and indirect effects were estimated according to LeSage [53], and statistical significance according to Dubin [54] through Monte Carlo simulation (MCS); 1000 random shocks were performed at ε_m_. We analyzed the robustness of our estimates by varying the number of neighbors (k = 5, 8, 10, 12 and 15). Effects were calculated using the spdep library in the R x64 3.2.5 statistical package.

### Ethical approval

The project was reviewed and approved by the Committees of Research, Biosafety and Ethics of the INSP, Mexico (Research committee: CI- 099–2017, Ethics committee: 1366, Biosafety committee: exempt).

## Results

Before the creation of SP in 2003, the analyzed municipalities presented the following characteristics: a high percentage of the population lacked social security (75% on average) and lived in rural areas (60%); many belonged to indigenous groups (20%) and experienced social marginalization and economic inequality; the average fertility rate was 3 children; and 21% of women lacked any educational experience. Between 2001 and 2015, the municipalities witnessed a 38% increase in available hospital resources and equipment for maternal health care as well as a 92% increase in available resources for outpatient primary care (Table 2).

**Table 2:** Main socio-demographic characteristics of women of reproductive age and maternal health resources at the municipal level, Mexico.

N = 2,314	Mean and IC-95%
**Prior to *SP* (2000)**^a^	
Population without social security (%)^b^	74.8 [74.1,75.6]
Rurality (%)^c^	60.0 [58.5,61.4]
Marginalization index (%)	41.8 [41.1,42.5]
Gini coefficient	46.0 [45.8,46.3]
Fertility^d^	3.1 [3.1,3.2]
Population dispersion (number of inhabitants per km^2^)^e^	261.8 [213.4,310.2]
Indigenous population (%)^f^	20.0 [18.7,21.3]
Population with no schooling (%)^g^	19.2 [18.8,19.5]
Women with no schooling (%)	20.9 [20.5,21.3]
Men with no schooling (%)	17.3 [17.0,17.6]
Ratio of women/men with no schooling	1.3 [1.2,1.3]
**Available maternal health resources**^h^	
Δ% 2015–2001: Hospital resources and equipment	38.2 [32.6,43.7]
Δ% 2015–2001: Outpatient care resources	92.2 [61.4,123.0]

**Notes:** a: Popular Health Insurance or *Seguro Popular*

b: population without social security (%)

c: residents of rural areas (%)

d: number of children born to women aged 12 years and above

e: number of inhabitants per km^2^ at municipal level

f: population aged five years and older who speak an indigenous language (%).

g: population with no schooling (%)

h: multivariate index of available resources estimated through principal component analysis. The index of available hospital resources included hospitals, hospital beds, operating rooms and ultrasound equipment as well as physicians and nurses in contact with patients. The index of available outpatient resources included outpatient health units and delivery rooms, as well as physicians, nurses, and medical students in contact with patients. Information sources: National Institute of Statistics, Geography and Informatics (*INEGI*), National Population Council (*CONAPO*), information sub-system on births (*SINAC*), and National Health Information System (*SINAIS*).

**Fig 1:**
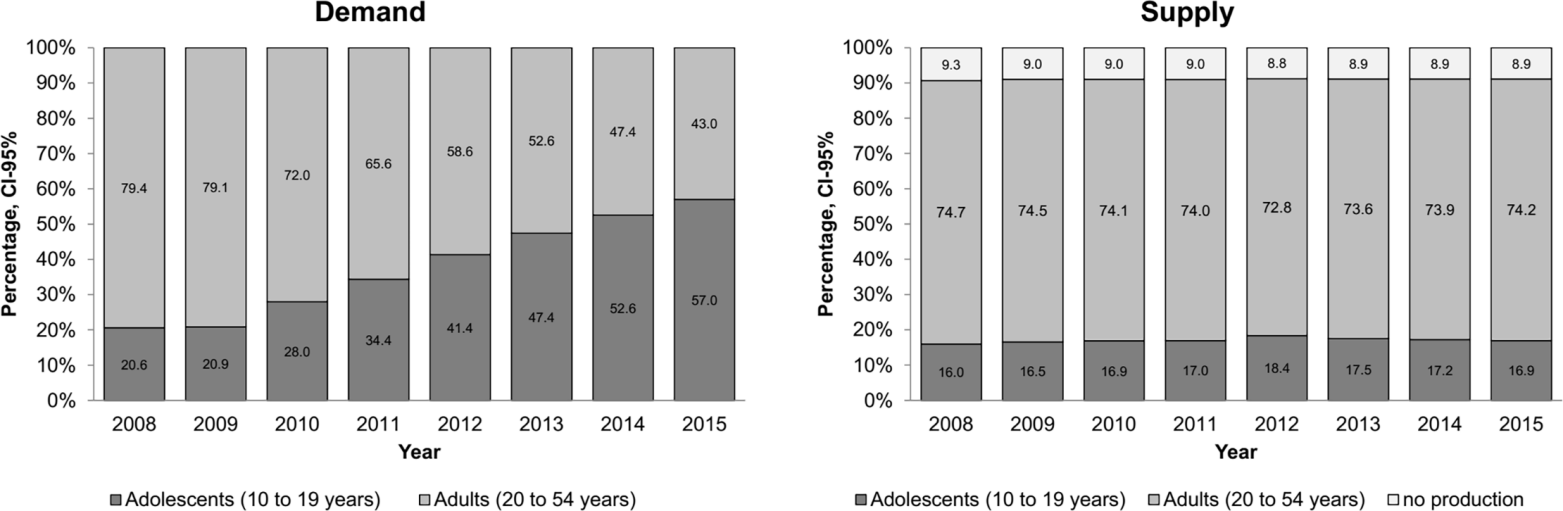
Evolution of municipal supply and demand for antenatal consultations (average %, 2008–2015), Mexico.

Fig 1 illustrates the evolution of supply and demand for ANC services between 2008 and 2015. As shown in this figure, the proportion of adolescent women vis-à-vis the total number of women requiring ANC rose sharply from 20% to 57%, whereas the distribution of the proportional first-time ANC consultations delivered by outpatient care facilities remained unchanged: 15% for adolescent and 74% for adult women.

**Fig 2:**
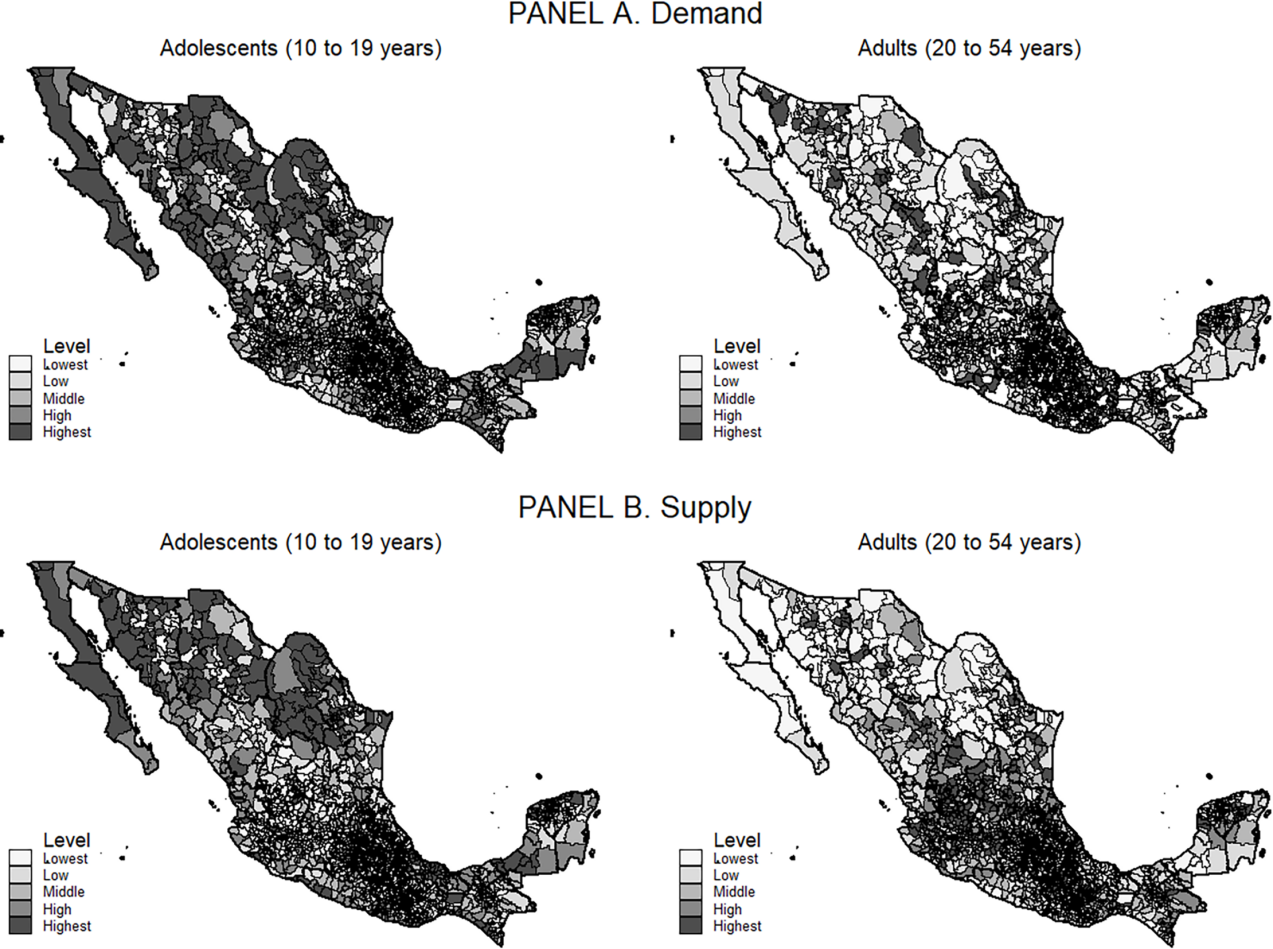
Supply and demand for antenatal consultations at the municipal level, Mexico, 2015. **Note:** Elaborated by the author. Public information sources: Information sub-system on births (SINAC) and National Health Information System (SINAIS). This figure was elaborated using the statistical program Stata MP v13.2.

In 2015, supply and demand for ANC services were territorially concentrated (Fig 2). The greatest demand for health services among adolescent and adult women occurred in the north and center of Mexico (panel A). Adolescent demand prevailed in Baja California, Baja California Sur, Coahuila and Sonora, while that of adult women prevailed in Guanajuato, Aguascalientes, Querétaro and Hidalgo. Notwithstanding the spatial distribution of demand, the largest supply of ANC consultations was registered in the north for both age groups (panel B). These facts clearly illustrate that demand and production were higher in the north of Mexico for adolescents and in the center of the country for adults.

**Fig 3:**
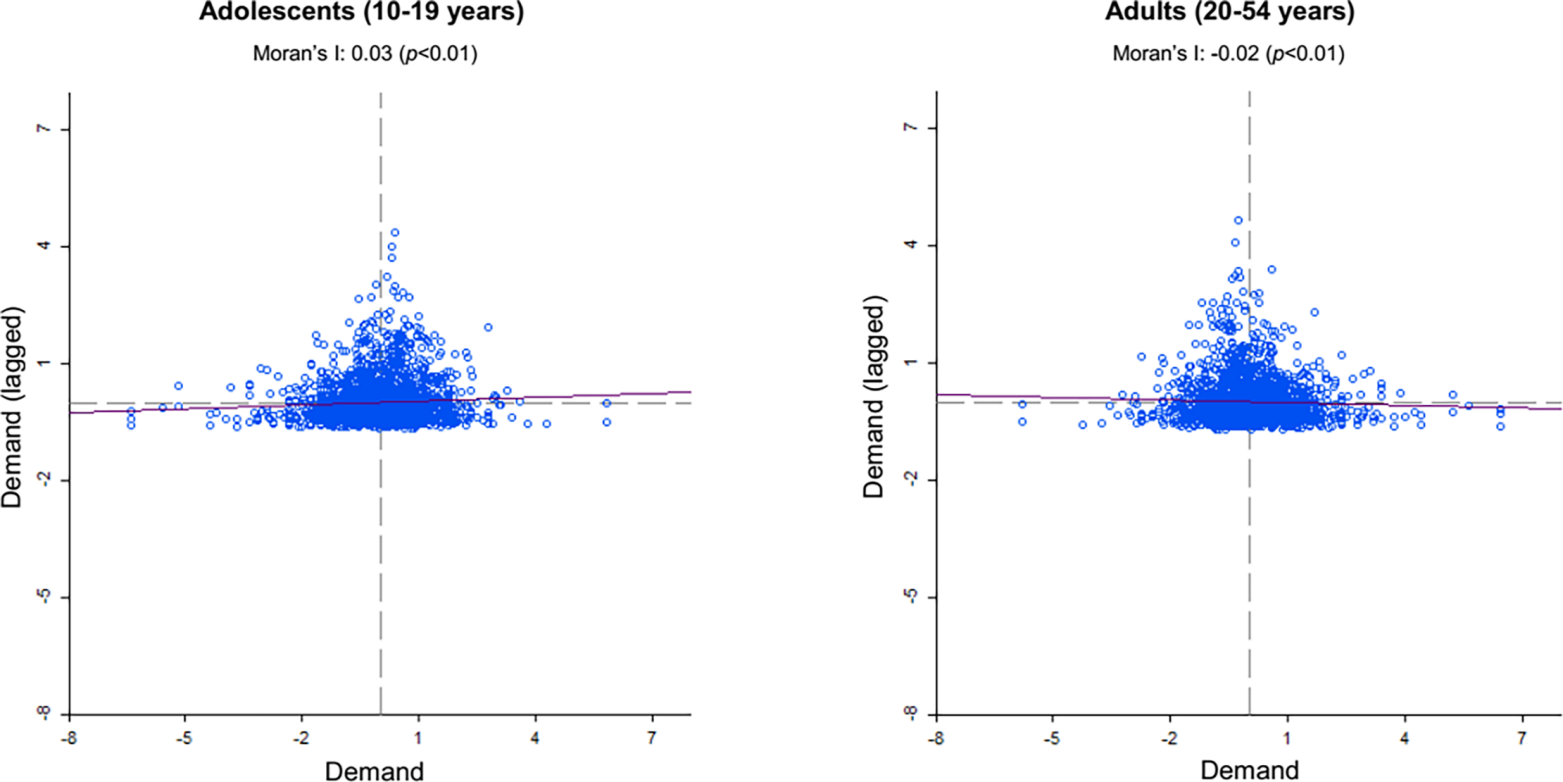
Bivariate spatial correlation between supply and demand for antenatal consultations at the municipal level, Mexico, 2015. **Note:** Estimates were based on the five nearest neighbors and included 10,000 shocks. Elaborated by the author using GeoDa.

Bivariate spatial analyses of correlation between supply and demand for ANC services yielded a minimal spatial correlation, or lack of territorial correspondence, between supply and demand among women in both age groups (Moran’s I/adolescents: 0.03, Moran’s I/adults: -0.03) (Fig 3).

Finally, the SDM confirmed a non-significant association between supply and demand for ANC services (Table 3). Sensitivity analyses performed with varying numbers of nearby neighbors (k = 8, 10, 12 and 15) supported the results’ robustness.

**Table 3:** Spatial Durbin model: Alignment between supply and demand for antenatal consultations at the municipal level, Mexico, 2015.

N = 2,314	Direct effect		Indirect effect		Total effect		Spatial lag parameters		Akaike information criterion (AIC)
	Impact	*p*	Impact	*p*	Impact	*p*	*ρ*	*θ*	
Adolescents (10–19 years)	-0.001	0.518	-0.002	0.581	-0.004	0.474	0.194	-0.002	17,653
Adults (20–54 years)	-0.118	0.139	-0.146	0.477	-0.264	0.257	0.198	-0.098	23,798

**Note.** The statistical significance of effects was obtained using Monte Carlo simulation (1,000 shocks). Estimates were performed by the authors using the spdep library in the R x64 3.2.5 statistical software package.

## Discussion

UHC is built on the provision of comprehensive health care services with financial protection. Access to sufficient and high-quality services should contribute to improving the health of the population. However, this positive association occurs when various conditions are met. Salient among them is a high degree of adjustment between supply and demand for health care services.

In a context of increasing access to essential health services in Mexico and sustained investments in maternal health, we assessed the degree of alignment of supply and demand for ANC among Mexican women without social security. The results of the econometric spatial analysis performed on administrative data from the MoH/SSa regarding ANC needs and service delivery show a misalignment between supply and demand in Mexico. In other words, the expansion of health care resources witnessed in recent years has failed to satisfy some of the health care needs of the population. In the maternal field, neither mothers nor their children are being provided optimal health care, while health care providers are missing the opportunity to identify and address the problems faced by women of reproductive age.

Health markets are characterized by imperfections (e.g., externalities and asymmetries of information) that result in market failure, an imbalance between supply and demand for health services [55–57], and, consequently, sub-optimal outcomes in health system performance [58,59]. The studies of access to specific health services have focused on the determinants of health service utilization, including personal profiles (age, ethnicity, occupation, schooling, income and insurance status), service barriers (geographic, financial, organizational and insurance-related), and availability of physical and human resources [60].

Following this tradition, we have studied the origin of ANC market distortions in Mexico, namely certain characteristics of the population (e.g., place of residence) and health care services (e.g., geographic location). We have found, for instance, that adolescent women, the population group with the greatest need for ANC services, struggle with the constraints of an insufficient production of timely antenatal consultations, especially in certain conglomerates. We have also observed that supply and demand for ANC present a territorial, univariate and non-random behavior: the demand for ANC services among adolescents is concentrated in the south and center of the country and that of adult women in the southwest and north, whereas production of ANC consultations for both population groups prevails in the north. In other words, supply and demand for these services correlate only to a minimum extent.

The main possible reasons for this misalignment, given the facts that maternal health is a national priority and there has been an important increase in resources for maternal health care for the population with no social security, include poor planning procedures, misallocation of resources (e.g., concentration of resources for maternal health in hospital settings), and/or corruption, which interact with the socio-demographic and territorial characteristics of municipalities. Further studies will be needed to measure the relative weight of these three potential causes in the lack of correspondence of supply and demand for maternal services. Further comparative analyses or qualitative studies at the state level should also be developed in order to identify and understand good and bad performers, and good and bad practices.

Misalignment in the maternal health market in Mexico also has international policy implications related to targets 3.1 (*By 2030*, *reduce the global maternal mortality ratio to less than 70 per 100*,*000 live births*) and 3.8 (*Achieve universal health coverage*, *including financial risk protection*, *access to quality essential health-care services and access to safe*, *effective*, *quality and affordable essential medicines and vaccines for all*) of the Sustainable Development Goals [61]. If the focus of health care services in outpatient units is not revised in accordance with national health care priorities and their effectiveness is not enhanced, these targets will not be achieved.

Our findings provide a useful lesson for developing countries: expanding public insurance is a necessary but not sufficient condition to achieve UHC of ANC services. The health care model and resource allocation should also guarantee access to geographically accessible, appropriate, sufficient, and timely services.

The methods we used in our study offer several advantages. First, they allowed us to analyze heterogeneity and spatial dependence in our study variables. Spatial heterogeneity stems from the characteristics of the units under observation. In our study, the characteristics of the sample municipalities were not homogeneous and consequently produced a heterogeneous relationship between supply and demand for services. Second, they also allowed us to correct problems that conventional econometric methods do not consider regarding spatial heteroskedasticity and spatial correlation in the data. This point is relevant as it is well known that phenomena tend to be alike among neighboring units and therefore generate spatial dependence. Finally, they allowed us to analyze territorially concentrated phenomena—in our case, unmet ANC needs—which, in turn, provide key evidence for designing targeted interventions.

It is important to mention that aligning supply and demand in the health market is a highly challenging task, because its components behave differently to those in the rest of the economy. In this complex market, public institutions, private enterprises seeking maximum profits, and non-profit organizations coexist, making it difficult to define an equilibrium price for allocating resources adequately. Moreover, demand in the health market is difficult to predict given the heterogeneity of its characteristics (Table 1); this is mirrored by the variability in the consumption patterns of the different population groups and areas. Regarding supply, factors other than those addressed in this study also feed into misalignment, namely the concentration of service supply in urban areas, and the differences in medical practice, quality levels, and resource efficiency. This clearly suggests the need for the government participation as a regulator in the healthcare market, and to focus its efforts not only on minimizing the effects of market failure, but also on ensuring equal opportunities through the redistribution of resources. In the case of maternal health care, these measures will strongly contribute to cover the needs of women seeking ANC.

Our study is subject to some limitations. First, with regard to the supply-demand relationship, factors other than the spatial aspects addressed here may be distorting the market, namely ANC service costs (e.g., waiting time), consumer preferences (for private or mixed public-private suppliers), information asymmetries (suppliers and demanders have access to different levels of information), inhomogeneous products (different quality consultations), and competition with other health priorities for resource allocation [62]. Second, since the analyzed data were drawn from an administrative source, under- or over-reporting of resources and consultations may be present. Either of these may have led to biased conclusions regarding supply, particularly if the distortion is associated with the characteristics of the facilities and/or municipalities. However, it is important to highlight that SINAIS data are processed and reported only after validation. Third, regarding potential bias on the demand side, utilization of ANC services can also be influenced by individual, cultural, and community characteristics. Use of municipal-level information may lead to an ecological fallacy, limiting the ability to draw causal inferences on an individual basis. Fourth, there is a potential endogeneity problem in the supply-demand relationship. Nonetheless, this problem is mitigated for the following reasons: (a) service supply was measured in 2015, but demand was defined as the 2000–2015 percent change in the number of individuals (aged 10–19 and 20–54 years) without social security who had given birth to at least one live-born child, thus capturing the demographic evolution of service demand; (b) the control covariables of the supply-demand relationship (except those related to available health resources measured under the percent change 2001–2015) referred to the year 2000, that is, three years prior to the creation of the SP; and (c) spatial dependence in the error term was incorporated into the econometric models, thus correcting any bias or inefficiency in the estimated parameters through the introduction of a spatial correlation process. A fifth and final limitation relates to the fact that the spatial structure selected may have influenced the estimated effects. In response to this constraint, however, we tested our estimates for robustness by modifying the number of nearest neighborhoods and obtained no significant variation.

In sum, our results suggest that misalignment exists between service supply on one side, and the needs of the population and the consumption patterns of ANC services of those without social security, on the other. The supply-demand gap identified by this study is potentiated by local structural conditions and by elements pertaining to the coordination of health market agents, but above all, by the age structure of the population and by the unavailability of health resources at the community level. It is therefore of primary importance to promote actions that facilitate access to ANC services in the regions where health needs are not being met. This will redound to an improvement in health system performance and to greater social wellbeing.

## Supporting information

S1 Table Spatial autocorrelation tests. (DOCX)

S2 Table Tests performed to corroborate nesting of the spatial error model in the spatial Durbin model. (DOCX)
