# Supplementary material for: Old principles, persisting challenges: Maternal health care market alignment in Mexico in the search for UHC
Source: PLoS One. 2018 Jul 2;13(7):e0199543. doi: 10.1371/journal.pone.0199543 (PMC6028103; doi:10.1371/journal.pone.0199543)
Supplement: S1 Table — (DOCX) [file pone.0199543.s001.docx]

**S1 Table**

Spatial autocorrelation tests

| **Test** | **Adolescents**  **(10-19 years)** | |  | **Adults**  **(20-54 years)** | |
| --- | --- | --- | --- | --- | --- |
|  | **Test statistics** | ***P*** |  | **Test statistics** | ***P*** |
| **Panel A:** k=5 |  |  |  |  |  |
| Spatial error: |  |  |  |  |  |
| Moran’s I | 21.7 | 0.00 |  | 19.6 | 0.00 |
| Lagrange multiplier | 454.7 | 0.00 |  | 372.3 | 0.00 |
| Robust Lagrange multiplier | 188.7 | 0.00 |  | 152.4 | 0.00 |
| Spatial lag: |  |  |  |  |  |
| Lagrange multiplier | 327.0 | 0.00 |  | 259.3 | 0.00 |
| Robust Lagrange multiplier | 61.1 | 0.00 |  | 39.3 | 0.00 |
| **Panel B:** k=8 |  |  |  |  |  |
| Spatial error: |  |  |  |  |  |
| Moran’s I | 24.2 | 0.00 |  | 22. 1 | 0.00 |
| Lagrange multiplier | 562.5 | 0.00 |  | 466.5 | 0.00 |
| Robust Lagrange multiplier | 276.8 | 0.00 |  | 233.9 | 0.00 |
| Spatial lag: |  |  |  |  |  |
| Lagrange multiplier | 361.7 | 0.00 |  | 278.3 | 0.00 |
| Robust Lagrange multiplier | 76.0 | 0.00 |  | 45.7 | 0.00 |
| **Panel C:** k=10 |  |  |  |  |  |
| Spatial error: |  |  |  |  |  |
| Moran’s I | 25.1 | 0.00 |  | 23.3 | 0.00 |
| Lagrange multiplier | 600.3 | 0.00 |  | 517.2 | 0.00 |
| Robust Lagrange multiplier | 317.8 | 0.00 |  | 281.2 | 0.00 |
| Spatial lag: |  |  |  |  |  |
| Lagrange multiplier | 360.4 | 0.00 |  | 282.8 | 0.00 |
| Robust Lagrange multiplier | 77.9 | 0.00 |  | 46.8 | 0.00 |
| **Panel D:** k=12 |  |  |  |  |  |
| Spatial error: |  |  |  |  |  |
| Moran’s I | 25.5 | 0.00 |  | 23.7 | 0.00 |
| Lagrange multiplier | 616.2 | 0.00 |  | 531.6 | 0.00 |
| Robust Lagrange multiplier | 347.6 | 0.00 |  | 309.1 | 0.00 |
| Spatial lag: |  |  |  |  |  |
| Lagrange multiplier | 342.3 | 0.00 |  | 266.2 | 0.00 |
| Robust Lagrange multiplier | 73.6 | 0.00 |  | 43.7 | 0.00 |
| **Panel E:** k=15 |  |  |  |  |  |
| Spatial error: |  |  |  |  |  |
| Moran’s I | 27.0 | 0.00 |  | 24.1 | 0.00 |
| Lagrange multiplier | 681.4 | 0.00 |  | 544.2 | 0.00 |
| Robust Lagrange multiplier | 412.6 | 0.00 |  | 333.4 | 0.00 |
| Spatial lag: |  |  |  |  |  |
| Lagrange multiplier | 344.1 | 0.00 |  | 257.4 | 0.00 |
| Robust Lagrange multiplier | 75.3 | 0.00 |  | 46.6 | 0.00 |
