# Supplementary material for: Old principles, persisting challenges: Maternal health care market alignment in Mexico in the search for UHC
Source: PLoS One. 2018 Jul 2;13(7):e0199543. doi: 10.1371/journal.pone.0199543 (PMC6028103; doi:10.1371/journal.pone.0199543)
Supplement: S2 Table — (DOCX) [file pone.0199543.s002.docx]

**S2 Table**

Tests performed to corroborate nesting of the Spatial Error Model in the Spatial Durbin model

|  | **Adolescents**  **(10 to 19 years)** |  |  | **Adults**  **(20 to 54 years)** |
| --- | --- | --- | --- | --- |
| **Panel A:** k=5 |  |  |  |  |
| LR-chi2: | 50.0 |  |  | 40.2 |
| Prob > chi2 | 0.00 |  |  | 0.00 |
| **Panel B:** k=8 |  |  |  |  |
| LR-chi2: | 58.1 |  |  | 36.5 |
| Prob > chi2 | 0.00 |  |  | 0.00 |
| **Panel C:** k=10 |  |  |  |  |
| LR-chi2: | 59.8 |  |  | 40.9 |
| Prob > chi2 | 0.00 |  |  | 0.00 |
| **Panel D:** k=12 |  |  |  |  |
| LR-chi2: | 54.9 |  |  | 40.0 |
| Prob > chi2 | 0.00 |  |  | 0.00 |
| **Panel E:** k=15 |  |  |  |  |
| LR-chi2: | 57.8 |  |  | 45.9 |
| Prob > chi2 | 0.00 |  |  | 0.00 |
